# Characterization of Inhibitory Anti-Duffy Binding Protein II Immunity: Approach to *Plasmodium vivax* Vaccine Development in Thailand

**DOI:** 10.1371/journal.pone.0035769

**Published:** 2012-04-27

**Authors:** Patchanee Chootong, Tasanee Panichakul, Chongrak Permmongkol, Samantha J. Barnes, Rachanee Udomsangpetch, John H. Adams

**Affiliations:** 1 Department of Clinical Microbiology and Applied Technology, Faculty of Medical Technology, Mahidol University, Bangkok, Thailand; 2 Department of Science and Technology, Suan Dusit Rajabhat University, Bangkok, Thailand; 3 Department of Global Health Infectious Disease Research Program, University of South Florida, Tampa, Florida, United States of America; 4 Department of Pathobiology, Faculty of Science, Mahidol University, Bangkok, Thailand; Université Pierre et Marie Curie, France

## Abstract

*Plasmodium vivax* Duffy binding protein region II (DBPII) is an important vaccine candidate for antibody-mediated immunity against vivax malaria. A significant challenge for vaccine development of DBPII is its highly polymorphic nature that alters sensitivity to neutralizing antibody responses. Here, we aim to characterize naturally-acquired neutralizing antibodies against DBPII in individual Thai residents to give insight into *P. vivax* vaccine development in Thailand. Anti-DBPII IgG significantly increased in acute vivax infections compared to uninfected residents and naive controls. Antibody titers and functional anti-DBPII inhibition varied widely and there was no association between titer and inhibition activity. Most high titer plasmas had only a moderate to no functional inhibitory effect on DBP binding to erythrocytes, indicating the protective immunity against DBPII binding is strain specific. Only 5 of 54 samples were highly inhibitory against DBP erythrocyte-binding function. Previously identified target epitopes of inhibitory anti-DBPPII IgG (H1, H2 and H3) were localized to the dimer interface that forms the DARC binding pocket. Amino acid polymorphisms (monomorphic or dimorphic) in H1 and H3 protective epitopes change sensitivity of immune inhibition by alteration of neutralizing antibody recognition. The present study indicates Thai variant H1.T1 (R308S), H3.T1 (D384G) and H3.T3 (K386N) are the most important variants for a DBPII candidate vaccine needed to protect *P. vivax* in Thai residents.

## Introduction

*Plasmodium vivax* is a cause of morbidity and mortality in Thailand and other countries in South East Asia and worldwide about three billion people live at risk of infection by *P. vivax* [1], [2]. Current public health surveys indicates vivax malaria prevalence has been on the rise in Thailand and *P. vivax* now accounts for more than 50% of all malaria cases since 2000 [3], [4]. Approximately 50% of the cases are in the migrant population. Vivax malaria is widespread and still an important problem in Thai-Cambodia border and Southern parts of Thailand in the Malayan peninsula. It is important to note that a significant portion of malaria cases in Thailand occur among temporary migrant workers from bordering countries [5], which presents a major challenge to prevention and control of malaria in the resident population.

*Plasmodium* blood stages are responsible for clinical manifestation during infection. In *P. vivax* the blood stage preferentially invades reticulocytes expressing the Duffy Antigen Receptor for Chemokines (DARC) [6]. Parasite ligands, Reticulocyte binding proteins (RBPs) and Duffy binding protein (DBP), respectively, mediate these critical invasion preferences for *P. vivax* [7], [8], [9]. Initial interactions are believed to be mediated by RBPs, which are a complex heterogeneous multi-gene family whose cognate receptors are undetermined [9], [10]. DBP is the product of a single copy gene and is a member of the Duffy binding-like erythrocyte binding protein family (DBL-EBP) family, which are expressed in the micronemes and on the surface of *P. vivax* merozoites, and is associated with the decisive junction formation step during the invasion process [8]. It is this critical interaction of DBP with its cognate receptor DARC that makes DBP an important anti-vivax vaccine candidate.

The erythrocyte binding motif of DBP is in a 330-amino-acid cysteine rich domain, referred to as DBP region II (DBPII) or the DBL domain, and is the minimal domain responsible for binding to DARC on Duffy-positive human erythrocytes [10], [11]. DBPII is an important vaccine candidate since anti-DBPII antibody inhibits *in vitro* binding to DARC, reduces merozoite invasion of human erythrocyte and can confer protection against blood stage infection [12], [13], [14], [15]. However, the analysis *dbpII* alleles in field parasites showed that DBPII is hypervariable compared to other DBP regions. The polymorphisms occur frequently at certain residues in a pattern consistent with selection pressure on DBP, suggesting that allelic variation functions as a mechanism for immune evasion altering immune recognition of DBP and therefore might limit vaccine efficacy [16], [17], [18]. Understanding protective immunity against DBPII haplotypes common in vivax endemic area is necessary for finding strategy for vaccine design.

In Thailand, a previous study found a high rate of nonsynonymous polymorphism of *dbpII* alleles among 30 Thai isolates. The highest frequency of polymorphism was found in residues D384G, R390H, L424I, W437R and I503K [19]. The phylogenetic analysis of *dbpII* Thai *P. vivax* isolates demonstrated that most Thai isolates shared distinct alleles with *P. vivax* isolates from different geographical areas with some allele groups so far unique to Thailand [19]. Since DBPII polymorphisms among Thai isolates are extensive and some are unique, understanding naturally protective antibody against DBPII needs to be defined. In this study, we evaluated immune antibody activity directed against the most common Thai DBPII epitopes for their functional inhibition of DBPII.

## Results

### Naturally acquired responses to total (PvSE) and DBPII

**Figure 1:**
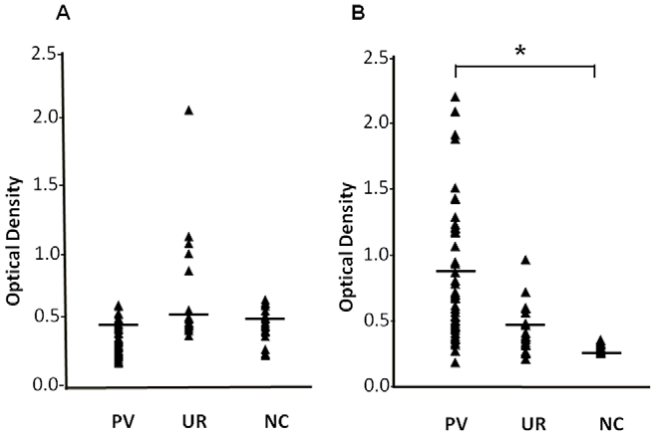
The antibody levels specific to *P. vivax* antigen. Graphical display of antibody levels anti-*P. vivax* shizont protein extract (A) and anti-DBPII (B) in Thai patients (PV), uninfected residents (UR) and naïve controls (NC). Dots represent the mean optical density value in triplicate wells for each sample. Bars represent mean value. Asterisk indicate statistic significant at P<0.05 with non-parametric two independent tests.

To assess the immunological responses during *P. vivax* infection, the reactivity of naturally acquired antibodies were tested against crude schizont antigen (PvSE) and the vaccine candidate DBPII. The anti-PvSE responses were very low in acutely infected *P. vivax* patients (average OD = 0.38±0.13), which had average antibody levels not significantly different from uninfected residents in the villages of the malaria endemic areas in Thailand (average OD = 0.44±0.25) and naïve controls (average OD = 0.38±0.14)(Fig. 1A). In contrast the antibody titer specific to anti-DBPII responses in individual patient's plasma samples were significantly elevated during *P. vivax* infections (average OD = 0.81±0.50) when compared with that of uninfected residents (average OD =  0.43±0.18) and naïve controls (average OD = 0.17±0.11)(Fig. 1B). In spite of this increased reactivity evident during vivax malaria infections, anti-DBPII responses of the Thai patients did not reveal any association between the parasitemia levels and the ages of patients (data not shown). The wide range of antibody responses to the recombinant DBPII antigen suggested a potential protective role of higher titer anti-DBP antibodies during *P. vivax* infection.

### The relationship between anti-DBPII response and the inhibitory function in Thai plasmas

**Figure 2:**
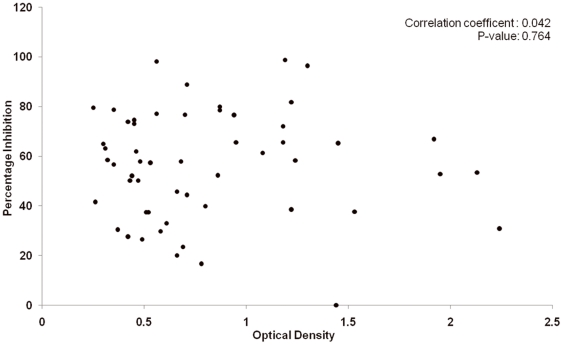
Anti-DBPII response and DBPII-Duffy positive erythrocyte inhibitory. Scatter plot showing the correlation between anti-DBPII levels and inhibition activity among Thai vivax residents (Spearman's coefficient: 0.042; P = 0.764). Fifty-four samples (1∶200 diluted plasma) were tested for their reactivity to DBPII in standard ELISA procedure and for the inhibition function of DBPII binding to Duffy positive erythrocyte measured by COS7 cell erythrocyte binding assay.

To further examine potential correlations with anti-DBP functional inhibition, anti-DBPII titers in individual patients were classified into 3 responder groups, high (HR), low (LR) and non-responders (NR). There were 15 samples in H group (OD value 2.24 to 1.08), 20 samples in L group (OD value 1.08 to 0.51) and 19 samples in N group (OD value less than 0.51), (Table 1). Inhibitory activity of Thai residents of *P.* vivax-endemic areas were evaluated to determine if their anti-DBPII levels correlated with functional inhibition of DBPII binding to human erythrocytes, (Table 1). The functional inhibitory efficiency of these samples to inhibit DBPII binding was determined using the *in vitro* COS7 erythrocyte binding assay and a wide range of DBPII inhibition was observed among Thai residents. Relatively few samples were completely inhibitory with only 3 samples of the HR group and 2 samples of the LR group at or near 100% inhibition (54 samples, Table 1). Most of the high anti-DBP titer samples had moderate to no anti-DBP inhibition. Importantly, anti-DBPII functional efficacy in Thai vivax patients did not correlate with anti-DBP titer (Spearman's coefficient: 0.042; P = 0.764)(Fig. 2).

**Table 1:** Naturally acquired anti-DBPII responses and their inhibition efficiency against DBPII binding.

Subjects	Age	Anti-DBPII Response (OD)^a^	Percentage Inhibition
1	19	HR	30.83
2	24	HR	53.41
3	23	HR	52.80
4	23	HR	66.90
5	20	HR	37.63
6	25	HR	65.23
7	48	HR	1.07
8	32	HR	96.42
9	25	HR	58.33
10	21	HR	81.77
11	50	HR	38.54
12	58	HR	98.90
13	38	HR	72.10
14	25	HR	65.60
15	20	HR	61.30
16	36	LR	65.59
17	20	LR	76.61
18	20	LR	78.53
19	30	LR	79.93
20	28	LR	52.30
21	27	LR	39.80
22	23	LR	16.67
23	21	LR	44.44
24	24	LR	88.89
25	39	LR	76.74
26	20	LR	23.50
27	19	LR	57.90
28	23	LR	19.98
29	21	LR	45.70
30	27	LR	32.98
31	30	LR	29.69
32	31	LR	98.20
33	23	LR	77.08
34	21	LR	57.35
35	21	LR	37.50
36	24	LR	37.50
37	27	NR	26.56
38	30	NR	57.90
39	48	NR	50.18
40	50	NR	61.93
41	20	NR	74.56
42	21	NR	73.10
43	24	NR	52.08
44	22	NR	50.18
45	25	NR	73.84
46	38	NR	27.60
47	36	NR	30.47
48	47	NR	78.70
49	20	NR	56.63
50	20	NR	58.43
51	28	NR	63.09
52	21	NR	64.90
53	27	NR	41.58
54	38	NR	79.50

a High responder (HR), Low responder (LR), Non-responder (NR).

### Anti-Thai DBPII epitopes inhibition of erythrocyte binding

**Figure 3:**
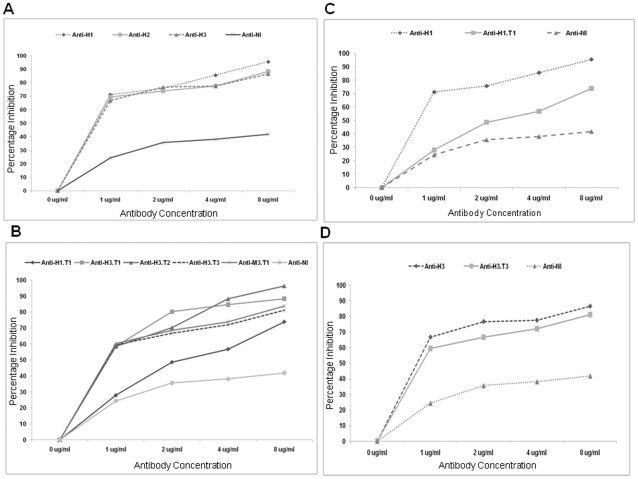
The inhibition activity of anti-DBPII epitope-specific antibody to Sal I and Thai strain epitopes. (**A**) Human antibody specific to DBPII epitopes strain Sal I; H1, H2, H3 and NI. (**B**) Antibodies specific to DBPII epitopes Thai strain; H1.T1, H3.T1, H3.T2, H3.T3, M3.T1 and NI. The purified antibodies were test to determine their inhibitory function against DBPII Sal I binding Duffy positive erythrocyte. The symbol indicates the mean percentage of inhibition of three experiments compared to the result of control experiment with no antibody. For antibody concentration of 8 ug/mL, the *P* value was <0.001 for comparison of H and NI peptides. Significance of inhibition was measured with the 50% inhibition concentration (1–2 µg/mL) of purified anti- H1, H2, H3 antibodies. (**C**) Anti-H1 compared with anti-H1.T1; (**D**) Anti-H3 compared with anti-H3.T3. Antibodies were tested to determine their inhibitory function against DBPII Sal I binding Duffy positive erythrocyte. The symbol indicates the mean percentage of inhibition of three experiments compared to the result of control experiment with no antibody. For antibody concentration of 8 ug/mL, the *P* value was <0.001 for comparison of H and NI peptides, and comparison H1.T1, H3.T3 and NI peptides.

To elucidate potential differences in DBPII epitope specificity and the preferred epitope targets for a protective vaccine candidate in the Thai population, we affinity-purified antibodies on peptides from defined neutralizing epitopes in DBPII. For this analysis we pooled samples identified as highly inhibitory, which were 3 high responder and 2 low responder samples. The anti-H1, H2, and H3 antibodies were tested for inhibitory activity against DBPII-Sal I binding to Duffy positive erythrocytes. The anti-H1, H2, and H3 affinity-purified antibodies showed significantly (P<0.05) stronger inhibition compared to purified antibodies specific to NI peptides (Fig. 3A). Inhibitory activity increased with the amount of neutralizing antibodies at 1, 2, 4 and 8 µg/mL. At 8 µg/mL of anti-H1, –H2 and –H3 had the highest inhibition, 95, 88 and 86%, respectively. Comparison of functional inhibition of total purified IgG from pooled inhibitory samples showed a more significant inhibition than total IgG of non-inhibitory samples (P<0.001, data not shown). Purified anti-H1, H2 and H3 rabbit sera also had an inhibitory effect against DBPII-Sal I binding but less than in the naturally-acquired inhibitory antibodies of Thai vivax patients (data not shown). Functional inhibitory activity of antibodies affinity purified to Thai DBPII epitopes (H1.T1, H3.T1, H3.T2, H3.T3 and M3.T1) was assessed by purification from pooled inhibitory plasma and tested using the COS7 assay. At 8 µg/mL of purified antibody had inhibitory function in range of 96–73%. Anti-H3.T2 had the strongest inhibition (96%) whereas anti-H1.T1 had the lowest inhibition (73%) against DBPII-Sal I binding to erythrocytes compare to purified antibody specific to other Thai DBPII epitopes (Fig. 3B).

To further assess impact of Thai DBPII polymorphisms to alter the specificity of an acquired antibody responses, we compared blocking function against Sal 1 DBPII of antibody affinity-purified on DBPII peptide of reference Sal I stain (H group) with Thai strain epitopes (Figs 3C, 3D). At 2 µg/mL, anti-H1.T1: FHSDITFRKLYLKRKL (49% inhibition) significantly decreased sensitivity of immune inhibition activity compare to anti-H1 Sal 1: FHRDITFRKLYLKRKL (76% inhibition, P<0.001)(Fig. 3C). Similarly, anti-H3.T1: GENAQQEEKQWWNESK (66% inhibition) had a inhibitory activity lower than anti-H3 Sal 1: DEKAQQRRKQWWNESK (76% inhibition) (Fig. 3D). The result suggests that the single or multiple mutations of amino acids (R308S, D384G and K386N) of Thai DBPII epitopes alter immune inhibition of DBP binding.

## Discussion

Antibody responses have an important role in protection against blood-stage *Plasmodium* infections. However, the complex blood stage cycle and the variation of target antigens hinder the effectiveness of humoral and cellular immune responses in defense against the parasite [20], [21], [22]. Naturally acquired anti-DBP antibody has the potential to block or inhibit parasite invasion [13], [15] and there is expected to be a boosting effect due to repeated exposure through recurrent infection [23]. *Plasmodium vivax* invasion requires the specific binding between *P. vivax* ligand, DBP and DARC receptor, making DBP a high priority anti-vivax vaccine candidate for malaria control. The characterization of naturally acquired anti-DBPII response is strain specific. The polymorphic nature of DBPII with each DBPII haplotype alters antibody recognition, and has an important role to evade host immune responses. The challenge of a DBPII vaccine is to overcome DBPII variation by understanding the nature of strain-specific anti-DBPII protective immunity. An effective DBPII vaccine will need to induce effective functional inhibition against all potential DBPII variants that circulate in malaria endemic areas. Here, we seek to identify variants that might influence efficacy of a DBPII vaccine in Thailand where there is low malaria transmission occurs with multiple-clone *P. vivax* infection. We first investigated the naturally acquired anti-DBPII response in individual Thai vivax patients and define the potential DBPII epitopes that could be protective vaccine candidate in Thailand.

Our study confirms the efficiency of naturally acquired anti-DBPII in protection against *P. vivax* infection previously demonstrated in Brazil and Papua New Guinea (PNG) vivax-endemic areas [12]. Anti-DBPII responses in acute Thai vivax patients were significantly higher than uninfected residents and their antibodies inhibited DBPII binding to Duffy positive erythrocytes. The result suggests the efficiency of naturally anti-DBPII in protection against *P. vivax* can have an active role in controlling *P. vivax* infections. The goal of DBPII-based vaccine development will be to elicit an antibody response against conserved epitopes that inhibits the adhesion of the DBPII ligand to its cognate erythrocyte receptor to block merozoite invasion of reticulocytes.

In the present study, we showed that the natural exposure to *P. vivax* in area of low unstable transmission of Thailand induced anti-DBP antibody that strongly inhibited DBPII binding. The inhibition activity of anti-DBPII in individual Thai residents did not show positive correlation with anti-DBPII responses and showed the wide range in inhibition activity. Two samples in low responder had a high inhibition and most high responders poorly inhibited DBPII binding. This is consistent with the study in PNG [14] and Brazil areas [12] in which anti-DBPII activity varied among vivax residents and also in another vaccine candidate, EBA-175, a wide range of functional antibody among lifelong resident of malaria holoendemic area in western Kenya [24]. To understand the variability of anti-DBPII inhibition, one possible explanation is the DBPII strain specificity through natural exposure of vivax infection. The target epitopes of anti-DBPII inhibitory antibody contains variant residues that can alter antigenic character and antibody recognition [25]. A longitudinal study to closely observe functional inhibition of anti-DBPII and B-cell memory response in transmission variation of Thailand will be required to determine the stability of naturally inhibitory anti-DBPII response.

The present study confirms the protective potential of previously identified H1, H2 and H3 epitopes in Thai residents. An important role of antibody responses to these epitopes is blocking DBPII-erythrocyte binding of by disruption of DBPII dimerization necessary for receptor binding [26]. Affinity-purified antibodies to the Thai DBPII peptides corresponding to the H1 and H3 epitopes showed significant differences in anti-DBPII inhibition against Sal 1 DBPII compared to the homologous Sal 1 antibodies. However, anti-H3.T2 inhibition still displayed high-level inhibition activity against DBPII-Sal I binding. The result suggests that certain mutations at the dimer interface, either monomorphic (R308S, D384K) or dimorphic mutations (D384K and K386N), can alter the efficacy of acquired neutralizing antibody recognition to block DBP function.

Anti-DBPII immunity in Thailand is induced by and targeting parasites in circulation in the endemic areas. The reference strain Sal I is not common, occurring in low frequency (10%), and is restricted to specific geographic areas [27]. We anticipate that the most effective strategy of DBPII vaccine will need to effectively target antigenically distinct DBPII variants common to the endemic areas. In Thailand, phylogenetic analysis showed Thai DBPII variants form a group with a subset of Papua New Guinea (PNG) and more related with Korea, India and Colombia isolates [19]. Interestingly, there are two haplotype unique among Thai *P. vivax.* The protective DBPII vaccine candidate in Thai resident will likely require antibodies directed against all DBPII epitopes of Thai DBPII variants in circulation among residents. Here, we identify Thai DBPII vaccine candidates that are associated with blocking antibody with Thai DBPII epitopes. The protective immunity of Thai vaccine candidate will require further characterization of Thai anti-DBPII responses to evaluate the most effective targets of blocking activity of antibody against Thai vivax isolates.

## Materials and Methods

### Ethics

This study was approved by the Committee on Human Rights Related to Human Experimentation, Mahidol University, and the Ministry of Health, Thailand (MU-IRB2009/300.012).

The participant information sheet was written and approved by Committee on Human Rights Related to Human Experimentation, Mahidol University, and the Ministry of Health, Thailand. The informed consent was singed by each individual participant before the blood sample (20 ml) was collected. The informed consent from minority participants, pregnant woman children <18 year were not involved in the study. Blood samples were collected from the acutely *P. vivax*-infected volunteers (n = 54), uninfected residents (n = 35) living at malaria clinic and from non exposed *P. vivax* donors (n = 40) healthy volunteers who live outside the endemic area and without previous history of malarial infection.

The selecting criteria of the patients were as followings: (1) systolic blood pressure was not less than 90 mm, (2) body temperature was not higher than 40^°^C, (3) Hematocrit was not less than 25% and (4) all patients have to be the age of 18 or above. Those who were not fitting the criteria were excluded.

### Study population

Blood samples were collected from the acutely *P. vivax*-infected volunteers (n = 54) at Malarial Clinics in Mae-Sod and Mae-Kasa districts, Tak province. Uninfected residents (n = 35) living at both districts were recruited in the study to compare antibody responses with vivax patients. In addition, samples from non exposed *P. vivax* donors (n = 40) healthy volunteers who live outside the endemic area and without previous history of malarial infection were included as naïve controls. The selecting criteria of the patients were as followings: (1) systolic blood pressure was not less than 90 mm, (2) body temperature was not higher than 40°C, (3) Hematocrit was not less than 25% and (4) all patients have to be the age of 18 or above. Those who were not fitting the criteria and pregnant patients were excluded. After the patients were selected, 20 ml of peripheral venous blood was collected. This study was approved by the Committee on Human Rights Related to Human Experimentation, Mahidol University, and the Ministry of Health, Thailand (MU-IRB2009/300.012). Informed consent was singed from each individual before the blood sample was collected.

### *P. vivax* cultures and *P. vivax* crude lysate antigens (PvSE) preparations

*P. vivax*-infected blood was used to prepare antigen, *P. vivax* schizont extract (PvSE). Briefly, *P. vivax* infected blood cells was depleted of white blood cell by filtering through a Plasmodipur® filter and the red blood cells were washed with RPMI-1640 by centrifugation at 1190×g for 5 min. The parasites were cultured for 24–30 hrs at 5% haematocrit in McCoy's medium (GIBCO, Carlsbad, USA) supplemented with 25% human AB serum at 5%CO2, 5% O_2_ and 90% N_2_. After the parasites had matured to schizont stage, antigens were separated by centrifuged in 60% Percoll^®^. The cells in the interface layer between medium and Percoll^®^ were collected, washed twice and the pellets were stored at−70^°^C to bed used for antibody detection.

### Measurement of antibody response to DBPII and PvSE

Anti-PvSE and anti-DBP responses were quantified by ELISA. The recombinant DBP regions II (rDBPII) was produced as described previously [14], [28]. Briefly, rDBPII was expressed as a GST fusion protein in *E. coli*, affinity purified on glutathione and cleaved from GST with thrombin using standard methods [28], [29]. Either 10 µg/mL of PvSE or 2 µg/mL of purified rDBPII-IV was added to 96-well plates, respectively, incubated for 30 min at room temperature, and washed three times with wash buffer (0.2% Tween-20 in PBS). Wells were incubated with 200 μL block buffer (2% skim milk in PBS) for 30 min, washed three times with wash buffer, allowed to dry and stored overnight at 4°C. Serum diluted 1∶400 in block buffer was added to pre-wetted wells and incubated for 90 min at 37°C. Plates were rinsed 3x in wash buffer, incubated with 1∶1000 goat anti-human IgG-alkaline phosphatase, rinsed 3x, and substrate added. Absorbance was recorded at 405 nm at 45 min after addition of developer reagent. A baseline was established using control sera from non-exposed Thai residents and this control value was subtracted from the test OD values. ELISA data was classified into three groups: High responders (OD = 2.24–1.05); Low responders (OD = 1.05–0.51); and Non responders (OD <0.51). The samples were considered positive when OD value is ≥mean +2SD of naïve controls.

### COS7 cell expression of DBP and inhibition assays

COS7 (green monkey kidney epithelial) cells [30] were transfected with the plasmid (pEGFP-DBPII-Sal 1), which allows expression of DBPII as a fusion protein to the N-terminus of EGFP used as a transfection marker as previously described [14]. The inhibition assay was performed 44 hrs after initial transfection. Serum at 1∶100 dilution or different concentrations of the affinity purified antibodies were incubated 60 min with transfected COS7 cells followed by incubation with Duffy positive human erythrocytes. Unbound erythrocytes were removed by washing three times with PBS. Binding was quantified by counting rosettes observed over thirty fields of view at 200x magnification. In this assay rosettes were defined as COS7 cells covered by bound erythrocytes at 50% or greater surface area. Percentage inhibition was calculated for each serum sample relative to binding in presence of non-exposed Thai residents control serum. Each assay included a duplicate test of each sample and results were determined from an average of 3 independent assays.

### Synthesized DBPII peptides

Mapping B-cell epitopes of naturally acquired antibody that block DBP binding to human Duffy positive erythrocyte in PNG residents showed H1: FHRDITFRKLYLKRKL, H2: EGDLLLKLNNYRYN and H3: DEKAQQRRKQWWNESK [25]. These epitopes contain polymorphic residues that may alter the antigenic recognition of antibodies. Therefore to define which peptides are the strongest DBPII vaccine candidate in Thai residents, the H1, H2 and H3 as well as Thai DBPII peptides which contain the most common alleles identified in the study Thai population [19] were synthesized. Therefore, eight DBPII peptides were synthesized (Table 2). The peptide purity was >90% as determined by high performance liquid chromatography.

**Table 2:** The synthesized peptides using in antibody purification and testing functional inhibition.

Peptides	Sequence
H1	FHRDITFRKLYLKRKL
H1.T1	FHSDITFRKLYLKRKL
H2	EGDLLLKLNNYRYN
H3	DEKAQQRRKQWWNESK
H3.T1	GEKAQQRRKQWWNESK
H3.T2	DENAQQRRKQWWNESK
H3.T3	GENAQQRRKQWWNESK
M3.T1	IEPOIYRRIREWGRDYVS
NI	CDGKINYTDKKVCKVP

H1, H2 and H3 are target epitopes of naturally acquired inhibitory antibodies [25]. H1.T1, H3.T1, H3.T2, H3.T3 and M3.T1 epitopes are the variant strain among Thai vivax isolates [19]. NI is the target epitopes of non-inhibitory antibodies.

### Antibody purification

Antibodies to B-cell epitopes were affinity purified from pooled human plasma containing high titer DBPII inhibitory antibodies from *P. vivax* exposed individuals of Thai or rabbit sera raised against peptides corresponding to synthesized B-cell epitopes. According to the manufacturer's recommended protocol diluted serum was passed over an affinity column prepared by coupling 3 mg of each peptide to a Sulfur Link coupling resin (Thermo scientific, Rockford, USA). After washing the column 3x with PBS, pH 7.4, the bound antibody was eluted with 0.1 M Glycine-HCL pH 3.0 and immediately neutralized with 1 M Tris-HCL, pH 8.5. Antibodies were dialyzed against PBS before storage at −20°C until needed.

### Data analysis

Classification of high-, low-, and non-responders to DBPII was based on averaged OD values for three wells per individual; a baseline was created from naive Thai plasma and subtracted from test OD values to standardize the ELISA [28]. Cluster analysis was performed on the ELISA values using SPSS (version 10.0); individual values clustered in these three distinct groups. High responders were defined as having OD values >mean +2 STD of Thai controls and Non-responder sample had OD values <mean +1 STD of the control plasma. Non parametric analysis (two independent samples; Mann-Whitney test) was used for comparison inhibition activity of purified anti-DBPII against H and NI peptides. The strength of association between percentage inhibition and anti-DBPII levels was analyzed by the non-parametric Spearman rank correlation coefficiency (*r*_s_).
